# Predictive models for health-related quality of life built on two telemonitoring datasets

**DOI:** 10.1371/journal.pone.0313815

**Published:** 2024-12-04

**Authors:** Matea Tashkovska, Stefan Krsteski, Emilija Kizhevska, Jakob Valič, Hristijan Gjoreski, Mitja Luštrek

**Affiliations:** 1 Department of Intelligent Systems, Jožef Stefan Institute, Ljubljana, Slovenia; 2 Faculty of Electrical Engineering and Information Technologies, Saints Cyril and Methodius University of Skopje, Skopje, North Macedonia; 3 Jožef Stefan International Postgraduate School, Ljubljana, Slovenia; Tehran University of Medical Sciences, IRAN, ISLAMIC REPUBLIC OF

## Abstract

Congestive heart failure (CHF) is an incurable disease where a key objective of the treatment is to maintain the patient’s quality of life (QoL) as much as possible. A model that predicts health-related QoL (HRQoL) based on physiological and ambient parameters can be used to monitor these parameters for the patient’s benefit. Since it is difficult to predict how CHF progresses, in this study we tried to predict HRQoL for a particular patient as an individual, using two different datasets, collected while telemonitoring CHF patients. We used different types of imputation, classification models, number of classes and evaluation techniques for both datasets, but the main focus is on unifying the datasets, which allowed us to build cross-dataset models. The results showed that using general predictive models intended for previously unseen patients do not work well. Personalization significantly improves the prediction, both personalized models and personalized imputation, which is important due to many missing data in the datasets. However, this implies that applications using such predictive models would also need to collect some self-reported labels of HRQoL to be able to help patients effectively.

## Introduction

Congestive heart failure (CHF) is a chronic condition that occurs when the heart’s ability to pump blood is impaired, often due to a combination of weakened cardiac muscles and increased stiffness. Although CHF can affect people of all ages, it is more prevalent among older adults. While there is no cure for CHF, its progression can vary widely among individuals, with some patients experiencing stable conditions for many years while others experiencing rapid deterioration [1].

Since CHF cannot be cured, it is important both to extend the patients’ survival and manage the quality of their life. While the former has always been a key medical objective, the latter is increasingly recognized by the medical establishment. As a result, Chiron and HeartMan [2, 3] both used mobile applications to collect daily self-reports on the feeling of health (FOH) on a five-point scale, which is a straightforward indicator of the patients’ health-related quality of life. The projects also included various technologies to monitor objective parameters related to health and quality of life (such as, blood pressure, heart rate, weight). Chiron primarily focused on telemedicine, while HeartMan aimed to help patients with the self-management of their disease. Both projects built machine learning models that predicted the FOH from the measured parameters. Chiron aimed to increase the understanding of the relation between these parameters and the FOH, while HeartMan also attempted to leverage the models to advise the patients how to improve their quality of life.

When studying predictive models in CHF, there are two key dimensions of interest: time scale (years, months, days) and target (morbidity/mortality, quality of life). Most CHF predictive models are long-term (years, months) and focused on cardiovascular disease events. Prominent recent examples are SCORE2 [4] and PREVENT [5]: the former was developed with 677 684 European patients from 45 datasets, while the latter was developed on 3 281 919 American patients from 25 datasets. To be able to develop models on such a multitude of datasets, it is necessary to use classical variables that are most commonly collected, such as blood pressure and cholesterol. These models rely on conventional statistics rather than machine learning. Some work also uses machine learning and a broader range of variables, for example a model built on UK Biobank data that was demonstrated to be more accurate than models using statistics or only classical variables. Predictive models on shorter time scales are less common, with the typical case being telemonitoring data used to predict worsening of the patient’s condition early enough that it can be managed effectively. One example is the HeartPredict algorithm [6], which was shown to tpredict hospitalizations in heart failure patients, improving on earlier approaches using simpler methods. Similarly, a model for predicting decompensation in heart failure patients [7], and a few additional examples can be found in a scoping review [6]. Both of these settings are similar to ours, but the prediction target is different. To the best of our knowledge, there are not many predictive models concerned with the quality of life. One example is the work by Allen et al. [8], who developed a simple predictive technique to identify individuals at high risk for poor quality of life in six months. There are also some more recent smaller efforts [9, 10]. Our previous work by Mlakar et al. [2] on the Chiron data showed that the telemonitored parameters contain useful information about the patient’s quality of life on a day-to-day basis. Similarly, the work on HeartMan data by Valič et al. [3] and Vodopija et al. [11] focused on improving quality of life on a day-to-day basis.

The first goal of this paper is to improve the accuracy of models to predict FOH from telemonitoring data compared to the models built in the Chiron and HeartMan projects. This was achieved by refining imputation and feature extraction methods. Our second and more important goal is to investigate whether such models are sufficiently general to be used by patients whose data was not used for training, and who may differ from those in training data in various unpredictable ways. To this end, we unified the Chiron and HeartMan datasets, which allowed us to build cross-dataset models. We then studied whether models for predicting the FOH generalize across patients and datasets, or whether they need to be personalized. We also investigated which features are important, and whether this generalizes across datasets.

## Methods

### Data

#### Data collection

The Chiron project included 38 CHF patients: 19 from the United Kingdom (UK) and 19 from Italy. However, some of the data were missing, therefore the research only included the information for 11 patients from the UK and 13 patients from Italy. There were 15 patients in New York Heart Association (NYHA) class II and 9 in NYHA class III. This is an established classification of CHF severity in which class II means mild symptoms and slight limitation during ordinary activity, while class III means marked limitation in activity due to symptoms, being comfortable only at rest. Table 1 presents the overall characteristics of the Heartman dataset. The primary device to measure their physiological parameters was a custom chest strap with a single-lead ECG monitor, a skin temperature sensor, a humidity sensor placed in the armpit, and an accelerometer [12]. To extract fiducial points from the ECG signal, the Falcon algorithm [13] was used. This allowed us to compute the heart rate as well as to describe each heart beat with additional parameters such as PR interval, QRS duration and QT interval. To track the activity, there was an additional accelerometer attached to the thigh. Based on the accelerometer data, an algorithm [14] was used to identify the patient’s physical activity. This algorithm further estimated the patient’s energy expenditure, which relates to the level of their activity. The data from the wearable sensors were sent to a smartphone. A smartphone app was used to record daily blood pressure, peripheral capillary oxygen saturation (SpO2), weight, ambient temperature and humidity. Patients’ observations lasted from 1 to 84 days.

The Chiron patients were asked about their FOH compared to the previous day. The answers available to them were as follows:

- 1 = much worse than yesterday
- 2 = somewhat worse than yesterday
- 3 = about the same as yesterday
- 4 = somewhat better than yesterday
- 5 = much better than yesterday

The 24 Chiron patients contributed a total of 1086 instances, where one instance consists of a set of sensor measurements accompanied by the self-report of the FOH.

Same as Chiron, the HeartMan clinical study was conducted across two countries: Italy and Belgium. 36 patients were involved in Belgium, and 30 patients were involved in Italy. The majority of the patients were classified as NYHA classes II and III. The overall population characteristics of the Chiron dataset are shown in Table 1. The primary device to measure their physiological signals was the HeartMan wristband, which collected the PPG signal, galvanic skin response, skin temperature, heart rate and RR intervals. The accelerometer in the wristband was used to extract the patient’s activity and energy expenditure [15]. The patients were also provided with a RuuviTag environmental sensor, which measured air pressure, temperature, and humidity. Also, both their weight and blood pressure were recorded daily through a mobile application. This application also guided patients through physical and relaxation exercises, and the record of these exercises is included in the dataset. The patients utilized the HeartMan system for three to six months providing a total of 749 instances.

The HeartMan patients were asked about their FOH compared to the usual one. The answers available to them were as follows:

- 1 = much better than usual
- 2 = somewhat better than usual
- 3 = about the same as usual
- 4 = somewhat worse than usual
- 5 = much worse than usual

**Table 1:** Overall population characteristics.

Population Characteristic	Chiron	HeartMan
Average Age (years)	62.9 ± 9.4	63±10.5
Sex	17 males, 7 females	24 males, 6 females
NYHA Class	II and III	II and III
Number of Days Participating	1 to 84 days	3 to 6 months
Total Number of Instances	1086	749
FOH range	1-5	1-5
Average FOH	2.93 ± 0.84	2.85 ± 0.93

**Fig 1:**
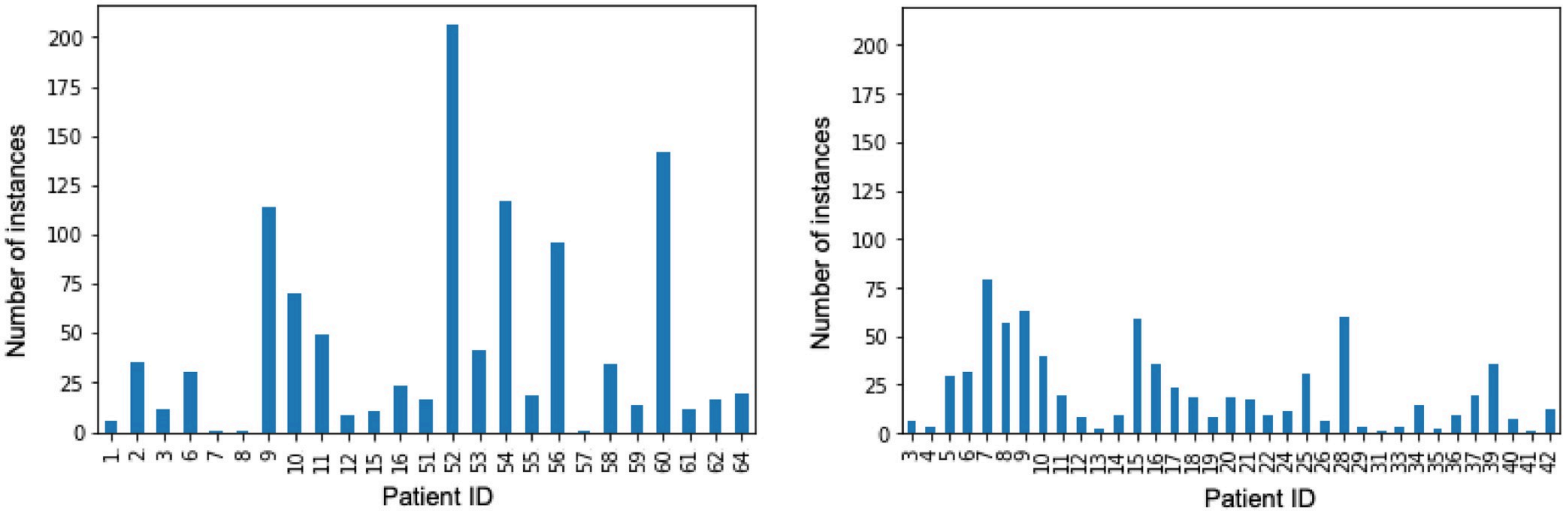
The distribution of feeling of health (FOH) self-reports for the Chiron and HeartMan datasets.

The distribution in Fig 1 shows the number of FOH instances per patient from both Chiron and HeartMan datasets. Some patients provided the information on their FOH every day, whereas some barely provided any.

For the Chiron data collection, the protocol was submitted to the ethics committes of Umberto I polyclinic in Rome, Italy, and to South East Scotland Research Ethics Committee in the UK. Ethics approval was obtained in each country, and the study carried out according to the national regulations. The participants were recruited between 1 January and 30 April 2013. They all gave written informed consent. The data collected in the study was already published [2] and retrieved for the purpose of this paper on 4 July 2022.

For the HeartMan data collection, ethics approval was obtained from the Central Ethical Committee of the University Hospital Ghent in Ghent, Belgium, and from the Central Ethical Committee of Lazio 1 of San Camillo-Forlanini Hospital in Rome, Italy. The participants were recruited between 1 January 2018 and 31 May 2019. They again all gave written informed consent. In both data collections, only the medical staff directly interacting with the patients knew their identity; the researchers analyzing the collected data received it de-identified. Raw sensor data was stored securely with strict access control.

#### Data transformation

**Fig 2:**
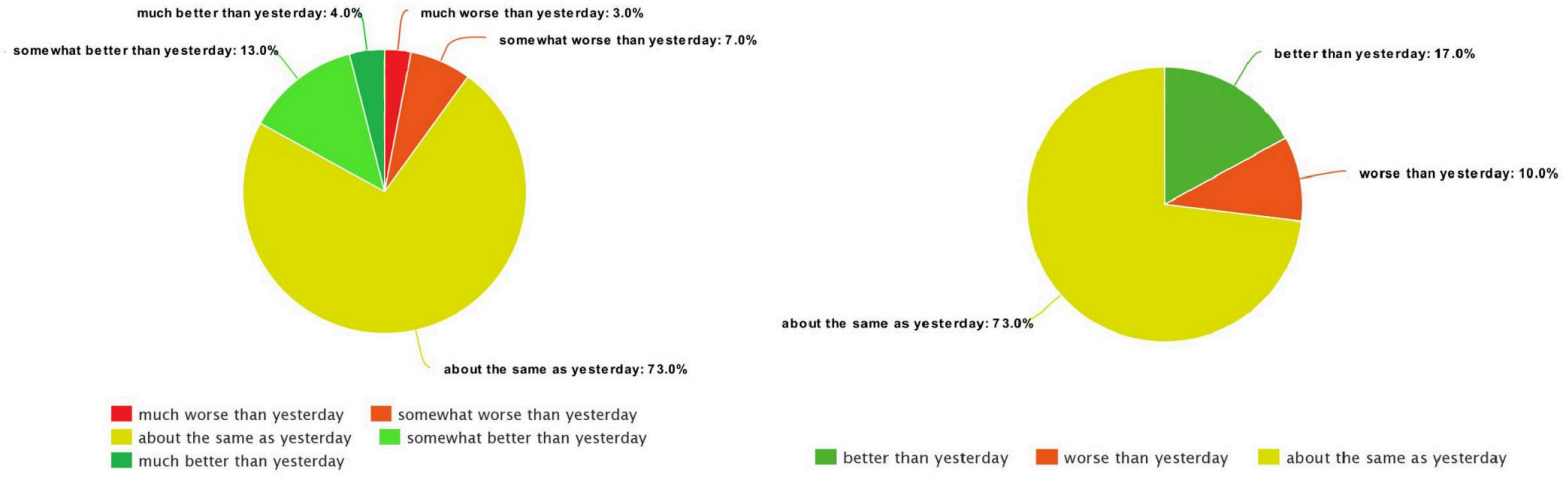
The class distribution for five (left) and three classes (right)—Chiron.

**Fig 3:**
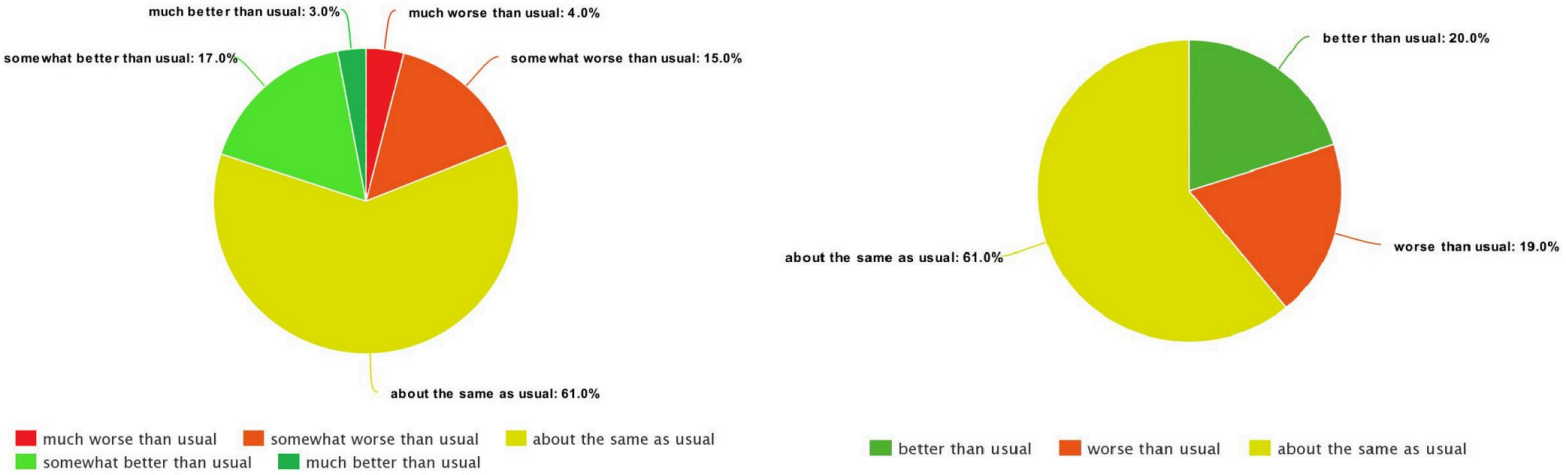
The class distribution for five (left) and three classes (right)—HeartMan.

As shown in previous papers [2, 3], it proved too difficult to distinguish between all five FOH classes. Also, using all five classes was causing a large class imbalance due to having few instances of extreme classes. Such an imbalance often results in models that predict the smaller classes poorly. Because of that, we merged 1 and 2, as well as 4 and 5, ending up with three classes. From Figs 2 and 3, we can see that this improves the class distribution. In some experiments we even removed the middle class, reducing the prediction to only two classes (better and worse).

In this study, we aimed to integrate the Chiron and HeartMan datasets to facilitate the training and testing of our models across both datasets. This integration required us to unify the classification schemas between the two datasets due to their differing approaches: the Chiron dataset labels daily health states relative to the previous day, while the HeartMan dataset labels them relative to the usual state of health. A stable state of health can be expected to correspond to stable physiological signals and environmental parameters, so it may lend itself better to modelling than comparative improvements in FOH. Additionally, we feel that achieving such a state is also a preferable objective for a practical application. Consequently, we adjusted the Chiron data to align with the HeartMan classes, which are based on this principle. This alignment was done following a set of rules:

1. Labels indicating a patient felt ‘same as yesterday’ were interpreted as ‘same as usual’. As this label was the most common, we inferred it generally represented the usual state. Similarly, ‘worse/better than yesterday’ was interpreted as ‘worse/better than usual’. This default rule applies unless superseded by one of the following more specific rules.
2. When ‘worse than yesterday’ was followed by ‘same as yesterday’, labels were changed to ‘worse than usual’ for up to five consecutive days starting from the ‘worse than yesterday’ instance. We reasoned that if ‘same as yesterday’ is reported shortly after worsening, it implies the patient continues to feel worse.
3. Consecutive days where a patient felt ‘worse than yesterday’ were left unchanged as these consistently indicated a ‘worse than usual’ state.
4. A label showing a patient felt ‘worse than yesterday’ immediately followed by ‘better than yesterday’ on the subsequent day was changed to ‘same as usual’ for that second day, reflecting an average baseline condition.
5. These transformations were applied only to consecutive reporting days. For gaps in daily reporting, no assumptions were made about the patient’s state on missing days, and labels from non-consecutive reports remained as originally recorded. No records were removed.
6. The same rules were applied symmetrically for ‘better than yesterday’ instances.

**Fig 4:**
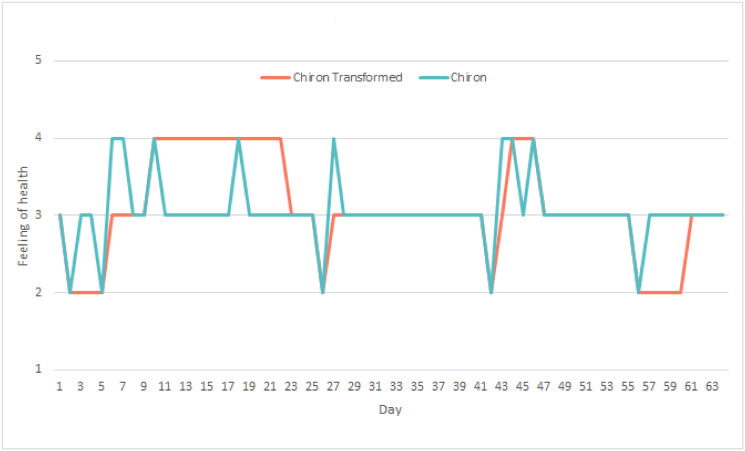
Example of the transformation of FOH in the Chiron dataset to align with the HeartMan labels, following the applied transformation rules.

The difference between the FOH in Chiron and its transformed version are shown in an example in Fig 4. This figure follows the transformation rules, and it shows the extent of the differences between the original and the transformed FOH.

### Feature extraction

Some of the features extracted from the Chiron and HeartMan datasets are similar, while others differ. These variations can be attributed to two main factors. Firstly, as detailed in the data collection section, the two studies utilized distinct sensor technologies, resulting in some unique features for each dataset. Secondly, the chronological development of the projects played a role. Chiron, being the earlier project, established a foundation of features. HeartMan, developed later, built upon this foundation and expanded the feature set, incorporating advancements and lessons learned from the Chiron study. This progression in development accounts for the additional features present in the HeartMan dataset.

#### HeartMan

We categorize the features into five distinct groups: discrete (e.g., blood pressure), continuous (e.g., heart rate), activity (e.g., the types of physical activities performed), exercise (related to the exercises guided by the HeartMan application) and environmental.

The discrete features consist of weight and blood pressure features. The weight features were calculated as two separate measurements: the change in weight from the previous day, and the change in weight over a four-day period. These are important because CHF patients tend to gain weight rapidly due to fluid retention. The one-day period was selected to align with the daily observation of FOH. The four-day period was recommended by a medical expert as a clinically significant timeframe in which a meaningful amount of fluid retention is likely to occur in heart failure patients. The same characteristics were calculated for systolic (SBP) and diastolic (DBP) blood pressure. Prior to feature calculation, we performed data curation on the weight and blood pressure values. This curation process involved identifying and addressing values that were physiologically highly improbable and likely resulted from data entry errors. We either removed these outliers or corrected them when possible, ensuring that our feature calculations were based on reliable data. For the SBP, we set an upper limit of 200 mmHg, and for the DBP, we set a lower limit of 40 mmHg.

Continuous features include skin temperature, galvanic skin response, and heart rate (HR). Here, data curation consisted of removing values below 40 bpm, which were typically due to poor sensor contact. We computed the rate pressure product (RPP) feature by multiplying the SBP by HR at 10-minute intervals before and after the recorded SBP value. RPP is used as a measure of cardiac workload, whose limitation is the major problem of CHF. Finally, we included two ratios: one between skin temperature and air temperature, and another between galvanic skin response and humidity. The rationale behind these ratios is that skin temperature is more informative when considered in relation to air temperature, rather than in isolation, and similarly, skin humidity is closely linked to galvanic skin response. These features were calculated for the intervals of 3 hours and 24 hours before the FOH entry, for the day of the FOH entry, and as the difference from the previous day (excluding the 3-hour interval for RPP, as blood pressure was typically measured only once a day). The rationale for using these intervals is that both short-term (3-hour) and long-term (24-hour) physiological states could contribute to the occurrence of FOH; sleep may act as a “reset,” which is why we included the day of the FOH entry; and changes over time (differences) may provide more insight than absolute values alone.

Each of the five activity features corresponds to the fraction of time walking, running, other movement, standing, and rest, measured during the day. The activity recognition was performed using a pre-built model [15] on the accelerometer data of 2-second intervals. The features were calculated for the day of FOH entry, the difference from the previous day, and for 24 hours and one week prior to the FOH entry. We included one week because activity over a longer period may affect how tired the patients are. Then, the ratio between the heart rate during the static activities (standing and rest) and the heart rate during the dynamic activities (walking, running, and other movements) was calculated. The energy expenditure was estimated using a pre-built model [15], taking into account the prevalent activity during the 10-second intervals. The rationale for these features is that a key symptom of CHF is the inability to be physically active, and the effort to be active may be reflected by heart rate during the activities.

The exercise features consist of the total number and the total duration of exercises done, and were calculated for the day of FOH entry and for one week prior to the FOH entry. The rationale for using a weekly period is similar to that of the activity feature, as the exercise program was organized in weekly segments. The exercise type (endurance or resistance) and the validity of the exercises was also used as a feature.

The environmental features consist of air humidity, air temperature and air pressure. These were calculated for the day of FOH entry and for 3 hours prior to the FOH entry.

For all the obtained features, except for the number of exercises and the recognized activity duration ratios, both the average and the standard deviation were calculated. Additionally, for all features except the exercise-related ones, the personal ratio was also computed. It is represented by each average feature divided by the average of all the values of that feature for the given patient, and each standard-deviation feature divided by the standard deviation of all values of that feature for the given patient. The rationale for personalizing the features is that how much a feature deviates from its typical value for a patient may be more indicative of their FOH than the absolute value of the feature. Personal ratios were also calculated for features representing the difference from the previous day. If data from the previous day was unavailable, we used data from the most recent available day, as long as it was within the last three days. For calculating the difference between the current weight and the weight from four days ago, if the four-day-old data was missing, we extended the search and used weight data from up to seven days ago to ensure the calculation was still meaningful despite potential data gaps.

#### Chiron

We grouped the features into two categories: features measured per day and personalized features.

The features measured per day include daily values, features measured multiple times during the day, and derived daily values. The daily values consist of ambient temperature, ambient humidity, SBP, DBP, SpO2, and weight. Body humidity, skin temperature, PR interval, QRS duration, T-wave amplitude, QT interval, HR, R-wave amplitude, and the patient’s energy expenditure were measured multiple times during the day. The derived daily features consist of the ratio between the average skin and ambient temperature, the ratio between the average skin and ambient humidity, RPP, double product (which is similar to RRP except that the avarege of SBP and DBP is used insteasd of SBP), the ratio between SBP and DBP, ratios between the duration of the three activities (lying, sitting, moving), differences between the average heart-rate values during all pairs of activities, ratios between the average heart-rate values during all pairs of activities, and the average ratio between the heart rate and energy expenditure. For every feature that was measured continuously or multiple times per day, the following features were calculated: the average value during the whole day, the average value during each activity (during lying, sitting, and moving), the standard deviation during the whole day and the standard deviation during each activity.

The personalized (normalized) feature values are represented as the ratio between the daily value of the parameter and its average value for the patient over the whole study period. Features that were personalized are: humidity ratio, temperature ratio, T-wave amplitude, R-wave amplitude, body temperature, body humidity, QRS, QT, and HR / energy expenditure.

### Preprocessing

Both datasets had many missing values, mostly because the patients did not consistently wear the sensing devices or enter the values manually. We thus chose a set of thresholds for the maximum permitted number of missing values for a feature. We removed all the features that had more missing values than each threshold, therefore, the more rigorous the threshold, the fewer features remained. The thresholds for Chiron were chosen based on previous research [2]. Best results were achieved when the features with more than 30% missing values were excluded. On the HeartMan dataset we experimented with different thresholds, where excluding the features with more than 60% missing values proved to be the best empirically. Predominantly, the excluded features were those related to exercise, activity, and their derived features for both Chiron and HeartMan.

Many features that were left still had missing values, so different imputation methods were used to solve this: k-nearest-neighbors (KNN) imputation using KNNImputer and multiple imputation by chained equations (MICE) using IterativeImputer, both from the Scikit learn library [16]. KNN imputes the missing values using the mean value of the same feature from the k nearest neighbors found in the dataset. On the other hand, MICE builds a model for each feature, that is then used to predict missing feature values using the available (non-missing) features as predictors. These methods are among the most popular and well-established techniques for handling missing data. KNN is suited for capturing local patterns in the data and MICE chosen for its ability to model complex feature interdependencies, allowing us to compare the different imputation strategies.

Also, two approaches were used with these imputation methods, global and personalized imputation. We developed personalized imputation because of differences in people, hypothesizing that imputing values based on data of the same person will work better than basing it of the full data. For example, for some people, a daily average heartbeat of 90 bpm might be normal, but this is not the case for everyone. This brought us to the conclusion that we should try imputing the values for each person individually. The dataset was divided into several subsets, each of which contained data from a single patient. However, after splitting the data, some of the patients had various empty feature vectors, so they could not be imputed using their own data. In such cases, the missing values were filled with the global mean.

Besides the already discussed features, we added an extra feature that represents the FOH from the previous day. After exploring the HeartMan data we saw a pattern that a person usually felt bad for a few days in a row. Because of the transformation, the same pattern was found in the Chiron-Transformed dataset as shown in Fig 4. So we assumed that a patients’ FOH from the previous day should correlate with their FOH. The difficulty was that the patients did not use the system consistently, leading to gaps of multiple days between entries in the majority of the subjects. When this was not the case, we used the FOH from the day before as a feature. Additionally, if there was no entry for FOH from yesterday, the field was filled with the class ‘same’. Should we wish to employ this feature in a practical system, it would have to ask the user about their FOH every day, which may be undesirable. Because of that, we treated this feature as optional.

### Model training

In the majority of machine-learning experiments, the classes were imbalanced, which can lead to models that do not work well for the underrepresented classes. To tackle this problem, we applied Synthetic Minority Oversampling Technique (SMOTE) [17], utilizing the imbalanced-learn library in Python, an approach in which artificial samples are created for the minority classes. We applied SMOTE exclusively to the training data to avoid leaking information to the validation phase and to maintain the integrity of the testing data. However, the synthetic samples may not fully capture the true distribution of the minority classes, which could potentially affect the model’s overall performance.

In our earlier work [11, 18], we compared several machine learning algorithms, including Random Forests, Support Vector Machines (SVMs), and Naive Bayes, where Random Forests consistently provided the best results. Random Forests are an ensemble method that aggregates the predictions of multiple decision trees, which makes them robust to variability in the data, an important factor in our study where sensor data can be noisy or incomplete. Additionally, the algorithm’s ability to handle both categorical and continuous data without the need for extensive preprocessing made it a suitable choice given the diverse feature set from the Chiron and HeartMan datasets. We used the scikit-learn library implementation of the Random Forest algorithm, with its default parameter values.

### Experimental setup

The evaluation techniques included 10-fold cross-validation (CV), leave-one-subject-out, and leave-half-a-subject-out.

- 10-fold cross-validation
  This approach randomly splits the data into 10 subsets. Then, the training is be repeated ten times, with each repetition conducted on a training set consisting of 9 subsets, and 1 subset used as a validation holdout set. This is the best-case scenario that is the easiest for machine learning, since data of each subject, sometimes from consecutive days, is found both in training and test data.
- Leave-one-subject-out
  The method known as leave-one-subject-out (LOSO) cross-validation uses each individual as a test set, while training on the other subjects. This corresponds to an application where the model is intended to be used by a subject who did not provide any personal labeled data.
- Leave-half-a-subject-out
  Leave-half-a-subject-out is a modification of the LOSO method to make it more personalized. Leave-half-a-subject-out is done by leaving half of each subject’s data out as the test set, and including the other half in the training set together with data of other subjects. Specifically, we use the first half of the data for training; for example, for days 0—100, days 0—50 are used for training and the remaining days for testing. This corresponds to an application where each subject provides some personal labeled data, e.g., for some days after he/she starts using the application.

As performance metrics, accuracy and F1-score were used. Accuracy can appear high even if just the majority class (’same’) is classified well. F1 weights all the classes the same and that is why it is given more importance than accuracy in our paper. Accuracy represents the number of correctly predicted instances divided by the total number of instances, shown as Eq 1.

$$\begin{array}{c} \text{Accuracy}=\frac{\text{Correct}\;\text{Predictions}}{\text{Total}\;\text{Predictions}} \end{array}$$ (1)

F1-score is defined as the harmonic mean between precision and recall, where precision is calculated by dividing the true positives by anything that was predicted as a positive, and recall is calculated by dividing the true positives by anything that should have been predicted as a positive. The F1-score is calculated with Eq 2. In the multi-class and multi-label case, this is the average of the F1 score of each class with weighting depending on the average parameter. We decided not to use ROC curves because our analysis involved both binary and multi-class outcomes. Using F1-score and accuracy ensures a more uniform and comparable evaluation across all classifications, making these metrics more suitable for our study’s varied data structure.

$$\begin{array}{c} F1=\frac{2\cdot \text{Precision}\cdot \text{Recall}}{\text{Precision}+\text{Recall}} \end{array}$$ (2)

These are commonly used evaluation techniques for classification. Accuracy can appear high even if just the majority class (’same’) is classified well. F1 weights all the classes the same and that is why it is given more importance than accuracy in our paper.

To summarize, we collected and processed data from the Chiron and HeartMan projects, extracting a wide range of features from wearable sensors and self-reports of FOH. We addressed missing data through personalized and global imputation approaches, transformed the Chiron dataset to align with HeartMan, and used various feature engineering techniques to capture both short-term and long-term physiological patterns. Machine learning models were trained using Random Forests, and multiple cross-validation methods were applied to ensure robust evaluation. We now turn to analyzing the results across various experiments to present the main findings of our work.

## Results

We start by comparing personalized and global imputation since the choice of the imputation approach affects all further experiments. Afterwards we study the impact of Chiron data transformation and the number of classes predicted across different types of cross-validation (10 fold cross-validation, leave-half-a-subject-out, leave-one-subject-out). We also check what happens if we add the FOH from the previous day to the features. We then perform cross-dataset experiments, Finally, we look at he importance of the different features for prediction.

### Imputation approach

The results for personalized and global imputation are shown in Table 2. We can see that personalized imputation significantly improves the F1-score in the HeartMan dataset. To ascertain if the observed difference between the means of global and personalized imputation is statistically significant, we conducted a t-test. We set the null hypothesis that there is no significant difference between the means, with a significance threshold of 0.05. The t-test resulted in a p-value of 0.042, indicating a significant difference between the two methods. This demonstrates that personalized imputation, which utilizes individual-specific data, leads to more precise handling of missing values compared to the global imputation. However, this is not the case for either Chiron or Chiron-Transformed, where the global imputation achieves a higher F1-score compared to the personalized. This was also ascertained using a t-test, resulting in a p-value of 0.004 for Chiron and 0.001 for Chiron-Transformed, which suggests a significant difference in the means. These results indicate that for these datasets, global imputation is more effective than personalized imputation in improving the F1-scores. The reason for this is difficult to identify. As a result, in all further experiments, the missing values for Chiron and its transformed version are filled using global KNN imputation, while in HeartMan they are filled using personalized KNN imputation.

**Table 2:** F1-score on three different datasets, for three classes, with global and personalized imputation using 10-fold CV.

		HeartMan	Chiron	Chiron Transformed
KNN imputation	Global	0.59±0.04	0.58±0.03	0.64±0.03
	Personalized	0.70±0.03	0.49±0.04	0.57±0.01
MICE imputation	Global	0.65±0.03	0.58±0.01	0.63±0.04
	Personalized	0.70±0.01	0.46±0.04	0.59±0.01

### Data transformation and type of cross-validation

In the previous subsection we can see that the transformed dataset provides better results. For further investigation, we made a thorough comparison between the two. The average accuracy and F1-score for experiments made on Chiron and Chiron-Transformed are shown in Table 3. When using three classes, Chiron has a higher accuracy than Chiron-Transformed. However, the accuracy—computed according to Eq 1—gives a greater weight to classes that are more frequent in the dataset. The majority class (’same’) is represented with 73% in Chiron, meaning that a majority model (that would always predict ‘same’) would have the acuracy of 73%. Compared to this, our three-class models are 3 percentage points (p. p.) better, 1 p. p. worse and 4 p. p. worse, depending on the evaluation setup. The majority class in Chiron-Transformed is represented with 67%, and the models are 7 p. p. better than the majority model, 4 p. p. better and 7 p. p. worse. Excluding the leave-one-subject-out model, which underperforms compared to the majority model, Chiron-Transformed demonstrated better performance. The F1-score—computed according to Eq 2—weighs all classes equally is is thus not affected by the overrepresentation of any class. Because it is important to correctly identify when the FOH is not ‘same’, we give greater weight to the F1-score than accuracy. F1-score is higher in Chiron-Transformed, confirming that the transformed labels are better related to the objective parameters from which they are predicted.

**Table 3:** F1-score and accuracy for two and three classes on Chiron and Chiron-Transformed, with three different CV approaches.

		Chiron		Chiron-Transformed	
		accuracy	F1-score	accuracy	F1-score
3 classes	10 fold cross-validation	0.76	0.59	0.74	0.66
	Leave-half-a-subject-out	0.72	0.55	0.71	0.62
	Leave-one-subject-out	0.69	0.31	0.59	0.28
2 classes	10 fold cross-validation	0.67	0.65	0.76	0.75
	Leave-half-a-subject-out	0.72	0.69	0.75	0.75
	Leave-one-subject-out	0.72	0.45	0.62	0.50

**Fig 5:**
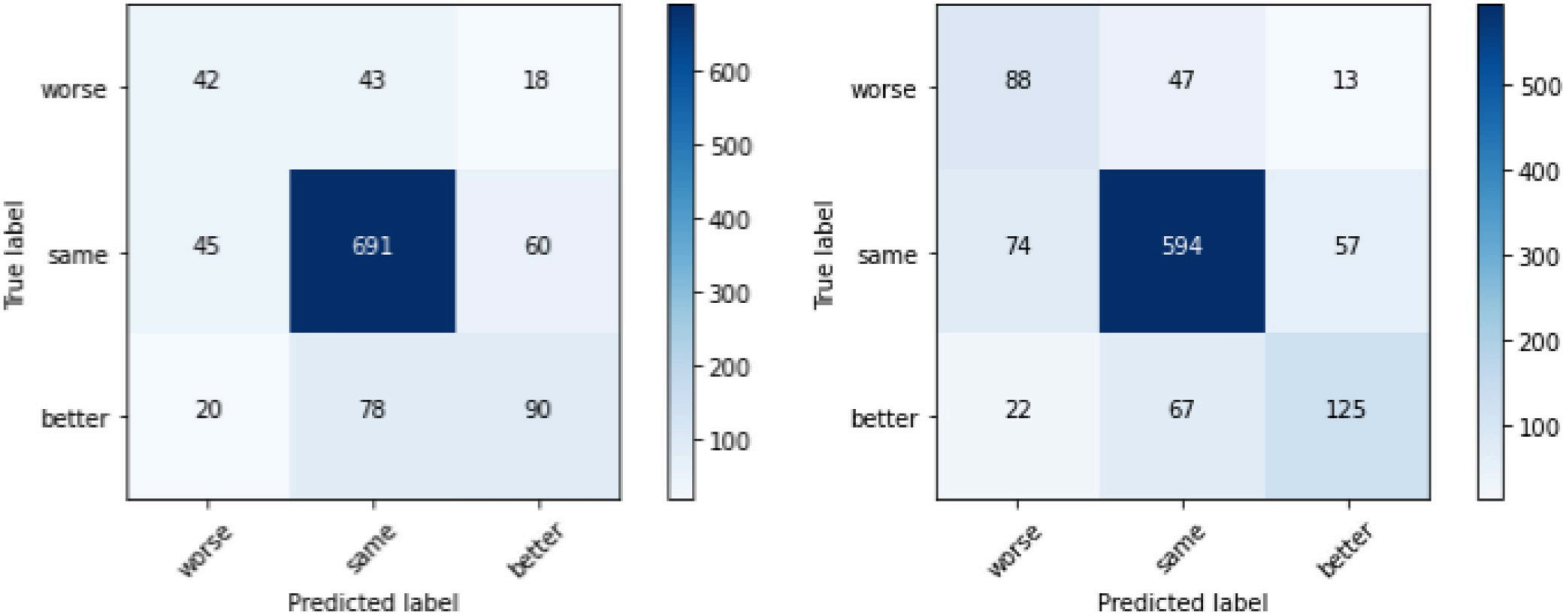
Comparison between the confusion matrices of Chiron and Chiron Transformed using 10-fold CV, with three classes.

The same thing is shown in Fig 5, which is a comparison between the confusion matrices of Chiron and Chiron-Transformed. As expected, the two models most accurately predict the class ‘same’. The confusion matrix shown in Fig 5 indicates that the model on the transformed dataset separates the FOH more clearly.

**Fig 6:**
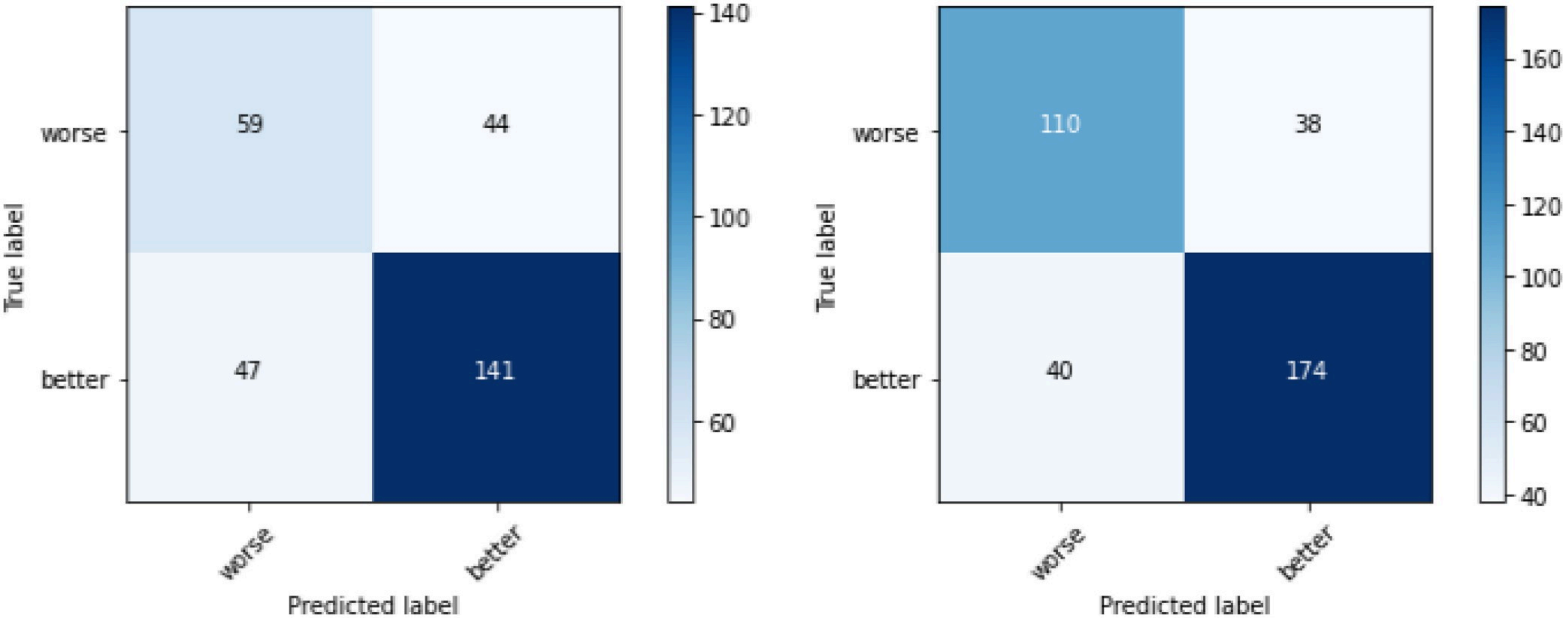
Comparison between Chiron and Chiron Transformed datasets using 10-fold CV, with two classes.

While there is a decent separation between ‘worse’ and ‘better’ with three classes, they are often confused with the class ‘same’, so we wanted to see how taking just the extremes works. After removing the class ‘same’, both the accuracy and F1-score are better for the transformed dataset in comparison to the original dataset, as shown in Fig 6. This again confirms that the higher accuracy in the experiment with three classes is a result of the class ‘same’. Even though the accuracy is probably the metric that is the easiest to grasp intuitively, it does not handle the overrepresentation of this class well, so the F1-score is used as the metric in all further experiments.

The same experiments that were done on the Chiron dataset were repeated on the HeartMan dataset (shown in Table 4). Once again, we can see the importance of personalization when we compare LOSO with leave-half-a-subject-out. 10-fold cross-validation is even better as even more data of test subjects is used for training. The best result is 90%, achieved with 10-fold cross-validation with two classes. This is an improvement in comparison to our previous study, which achieved accuracy of 57% for three and 68% for two classes using 10-fold cross-validation [3].

**Table 4:** F1-score for two and three classes on the HeartMan dataset, with three different CV approaches.

		HeartMan
3 classes	10-fold cross-validation	0.70
	Leave-half-a-subject-out	0.64
	Leave-one-subject-out	0.25
2 classes	10-fold cross-validation	0.90
	Leave-half-a-subject-out	0.85
	Leave-one-subject-out	0.30

**Fig 7:**
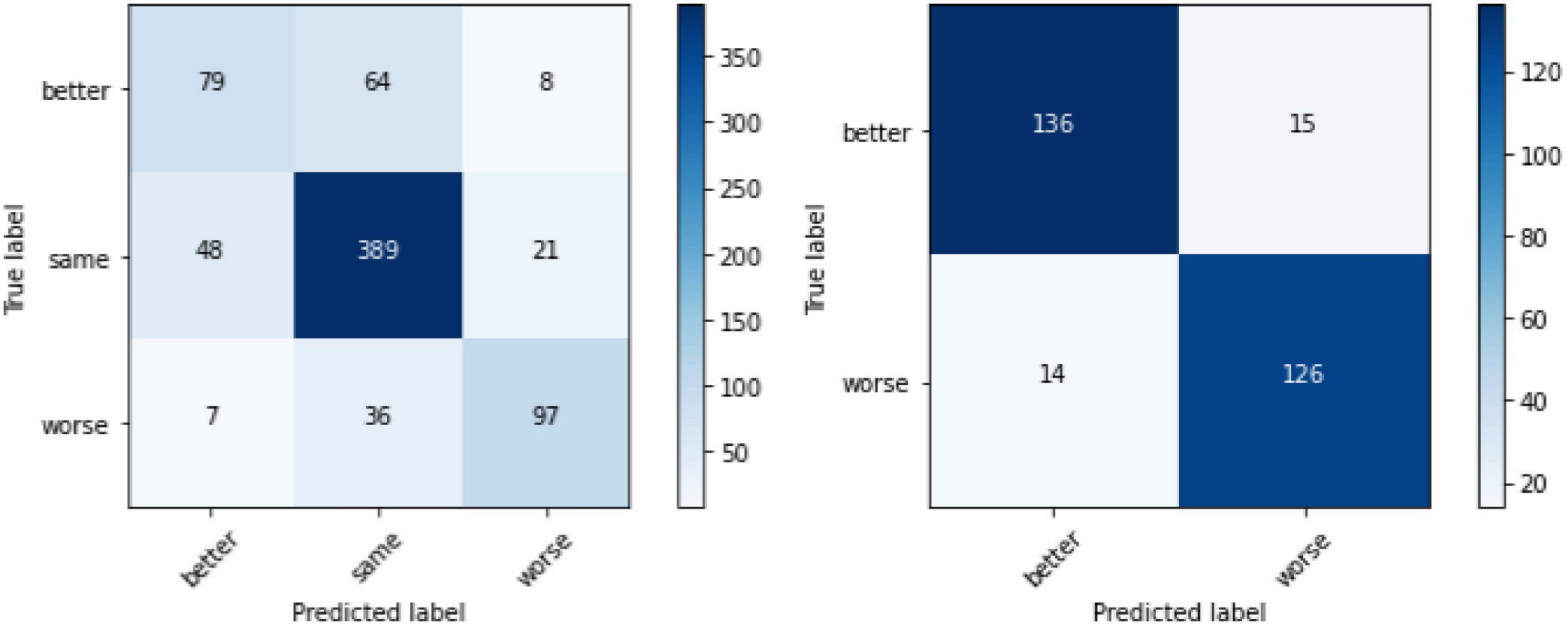
HeartMan confusion matrices for three and two classes using 10-fold CV.

Fig 7 represents HeartMan confusion matrices for three and two classes using 10-fold cross-validation. Predicting whether the patient is feeling worse is of higher importance, because this allows for better managing of the symptoms, thus improving the patient’s quality of life. In the three-class matrix, we can see that 97 out of 140 cases are correctly classified as worse. The results are even better after removing the class ‘same’, which is expected. The best results were achieved with 10-fold cross-validation on two classes, with an F1-score of 90%. However, the results achieved with LOSO are not satisfactory, which means that personalization is needed.

### Inclusion of FOH from the previous day

Table 5 shows the F1-score for both datasets with and without including the FOH from the previous day as a feature. Some improvement can be seen in Chiron-Transformed, meaning that knowing how the patient felt the previous day helps determine his FOH. Even though this makes sense, the results on the HeartMan dataset are unchanged. A possible reason for this is that the FOH entries were not as consecutive as the ones in Chiron, which caused the FOH from the previous day to be unknown in most of the cases. Another possible reason may be that while transforming the Chiron dataset we unintentionally forced it to be more dependent on the FOH from the previous day. Assuming that the results for the transformed dataset are biased, we decided not to include this feature in further experiments.

**Table 5:** F1-scores for experiments on HeartMan and Chiron-Transformed datasets with three classes, with and without FOH from the previous day, using leave-half-a-subject-out.

	HeartMan	Chiron-Transformed
With FOH from the previous day	0.64	0.67
Without FOH from the previous day	0.64	0.62

### Cross-dataset experiments

After determining which imputation works best, whether the transformed Chiron dataset is more appropriate than the original one, and whether FOH from the previous day should be used, we analyze how general our models are, and how compatible are the datasets.

In Tables 6 and 7, cross-dataset experiments are shown. The first two experiments consist of training on one dataset and testing on the same dataset including all features, as well as only including compatible features (common features from both datasets). Using compatible features only slightly reduces the accuracy in 10-fold cross-validation and leave-half-a-subject-out. So some of the incompatible features are valuable at least when partial personalization is possible. These results are already presented in the previous tables.

**Table 6:** F1-scores for cross-dataset experiments when testing on the Chiron-Transformed dataset.

Test on Chiron					
Train		Chiron, all features	Chiron, compatible features	Chiron + HeartMan	HeartMan
3 classes	10-fold cross-validation	0.64	0.62	0.62	0.27
	Leave-half-a-subject-out	0.62	0.62	0.60	
	Leave-one-subject-out	0.28	0.28	0.28	
3 classes	10-fold cross-validation	0.78	0.78	0.77	0.54
	Leave-half-a-subject-out	0.75	0.71	0.71	
	Leave-one-subject-out	0.5	0.4	0.52	

**Table 7:** F1-scores for cross-dataset experiments when testing on the HeartMan dataset.

Test on HeartMan					
Train		HeartMan, all features	HeartMan, compatible features	HeartMan + Chiron	Chiron
3 classes	10-fold cross-validation	0.70	0.61	0.60	0.32
	Leave-half-a-subject-out	0.64	0.58	0.55	
	Leave-one-subject-out	0.25	0.28	0.28	
3 classes	10-fold cross-validation	0.90	0.82	0.82	0.32
	Leave-half-a-subject-out	0.85	0.82	0.79	
	Leave-one-subject-out	0.30	0.37	0.41	

**Fig 8:**
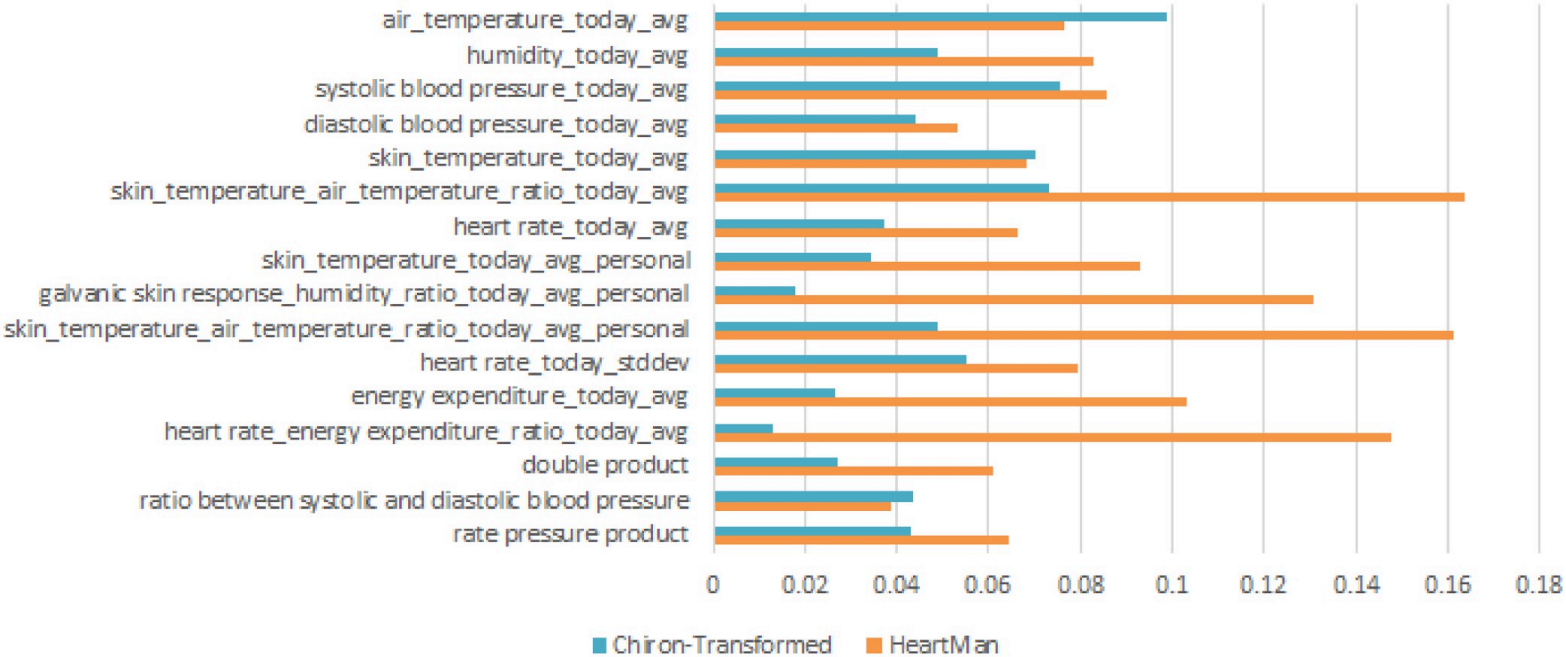
Feature importance across all compatible features present in the HeartMan and Chiron-Transformed datasets.

In the third experiment, the Chiron and HeartMan datasets were integrated prior to model training, using only the intersecting features. All features present in the two datasets and used in this experiment are shown in Fig 8. This was necessary to ensure compatibility between the datasets. Specifically, during the training and testing phases on each dataset, the other dataset was merged into the training data using these common features. Adding the other dataset to the training data decreases the F1-score in 10-fold cross-validation and leave-half-a-subject-out. This is probably because of the importance of personalization (as demonstrated in the previous experiments) as combining datasets dilutes the training data, so that it is less personalized.

The fourth experiment consists of training on one and testing on the other dataset. Only the features existing in both of the datasets were used, as shown in Fig 8. The results are similar to LOSO, so the datasets may be compatible enough, but the lack of personalization is the issue.

### Feature importance

Fig 8 shows the importance of all the compatible features, present in both datasets. for predicting the FOH, as calculated with the Random Forest built-in feature importance [19]. None of the features in either dataset were individually strongly correlated with the FOH, but the measure of feature importance used here takes into account their interaction with other features, so it can reveal important features that cannot be identified with simpler methods.

## Discussion

Building models for predicting health-related quality of life is interesting in order to understand what factors affect it, and perhaps even more to identify actions that improve the quality of life. Such models can be incorporated in personal health systems and clinical decision support to make patient- and context-specific suggestions for such improvement. A key finding of our analysis is that general models intended for previously unseen patients (LOSO) built on HeartMan and Chiron-Transformed are disappointing. This suggests that a practical application that would use such models would not work out of the box, and that personalization is crucial when working with patient-reported outcomes. For example, in Table 7 we can see that the leave-half-a-subject-out experiment (personalized model) for three classes has an F1-score of 64%, in comparison to LOSO which has an F1-score of 25%. The difference is even bigger if there are two classes, with percentages of 85% and 30% respectively. We also see that mixing datasets degrades performance, which means that a model built for a practical application should use data that is as similar as possible to the data arriving in practice. However, this problem is not as severe as lack of personalization and may be connected to the quality of data. As evidenced by Table 6, adding HeartMan data (which seems better) to Chiron caused little harm and in one case even an improvement.

We imagine that a model for a practical application should be built on datasets as close to the target application as possible. If no very similar datasets are available, a variety of datasets of sufficient quality may allow the model to handle different inputs, although our study does not provide direct evidence of this. This model should then be personalized, which means that the user would need to label some data. We believe that the best home for such a model would be a personal health system, probably in the form of a mobile application. Such an application would collect data from wearable and other monitoring devices (activity tracker with HR monitor, blood pressure monitor, scales, ambient sensors …). It would also, at least at the beginning, ask the patient about their FOH. This is undesirable, but—we believe—not a major inconvenience. The personal data about FOH would be used to personalize the model. Once personalized, the model would make a prediction about FOH daily, and if poor FOH is predicted, advise the patient how to improve it. This could be done using the method proposed in our earlier work [11], or some method based on counterfactuals, which would search for plausible patient states that are similar to the current one but have a better FOH [20]. A variant of the models discussed in this paper for longer-term FOH prediction could also be used in clinical practice, allowing health professionals to see what FOH is predicted for their patient, and what FOH would be predicted of certain changes to their health parameters are achieved. This would facilitate fine-tuning of treatment and advising the patient on disease management. Both at-home and clinical applications would improve the patients’ health-related quality of life, which is very important in an incurable disease such as CHF.

Another important part of personalization is the personalized imputation, which significantly improved the results on the HeartMan dataset. F1-score is 11 percentage points higher with personalized KNN imputation. Unfortunately, this is not the case for the Chiron dataset. A greater homogeneity of patients in the Chiron dataset could be an explanation, but the datasets appear quite similar in this respect. Since there is a lot of missing data in both datasets, and since CHF patients generally cannot be expected to provide data very diligently due to poor health and poor digital literacy, imputation is important in this domain, and we believe that doing it in a personal manner is a valuable innovation.

A question our analysis touched upon is how to pose the question of the FOH. The best F1-scores for the Chiron-Transformed dataset are 66% for three and 75% for two classes. This is an improvement of 7 percentage points for three and 10 percentage points for two classes, in comparison to the non-transformed Chiron dataset. This is true even though we included features characterizing differences from the previous day to match the original Chiron FOH formulation. So this might point to the fact that the question in Chiron was confusing. In the transformed version of Chiron, we changed the patient-reported FOH in such a way as to be in context with their usual feeling. This might add up to the personalization of the model, in a way that the term ‘usual’ is quite subjective.

As mentioned before, the results on HeartMan are better in comparison to our previous studies. The paper by Valič et al. [3] found the most satisfactory results to be 68% achieved with 10-fold cross-validation, for two classes. After they excluded the Italian data from the dataset, the accuracy went up to 76%. On the other hand, Vodopija et al. [11] achieved the best accuracy of 86.6% with 10-fold cross-validation, also for two classes. The best result in our paper for HeartMan is an F1-score of 90% achieved with 10-fold cross-validation for two classes. Personalization may be a big factor in this difference. Also, in this analysis, we extracted 238 features from the HeartMan dataset, as opposed to 72 features in the previous studies.

We showed the importance of features for the prediction of FOH in Fig 8. While conclusively determining why these features are important would require dedicated experiments, we can offer some hypotheses. The ratio features are the best features in the HeartMan dataset, possibly because they combine two relevant parameters in a meaningful way. However, they are worse in the Chiron dataset, possibly because the sensing hardware there was not as good, so the quality of the data was lower, and combining two parameters also introduces two sources of noise. Temperatures are the best features in the Chiron dataset. They were already observed as important in the earlier Chiron study, although a bit less than humidity and SBP features (which are also present in Fig 8). Good FOH is related with high and rising ambient temperature, which may be because CHF patients experience cold extremities due to poor blood circulation, and it is known that there is an increase in hospitalizations and mortality during cold weather [21]. Heart rate and energy expenditure are also important in the HeartMan dataset, which is expected as heart function and inability to be physically active are key elements of CHF. It is not clear why they are less important in the Chiron dataset.

As we are aware of no work except ours dealing with the exact problem tacked in this paper, we cannot directly compare these finding to related research. Closest to ours is the work on machine learning for prediction of serious worsening of health (leading to hospitalization). One observation we can make about these papers is that they all use only one dataset. This is probably because each telemonitoring system is different and there is no set of established variables to monitor, such as the classical risk factors that enable long-term risk prediction to pool multiple datasets. Blood pressure, HR, weight and SpO2 are monitored in most cases, but other variables are typically necessary to achieve satisfactory results, and these differ between datasets. It should be noted that validation on additional datasets is an issue even with long-term predictive models as noted in a review [22]. We also looked at the features that proved important in related research. Only one of the papers we identified as relevant—by Kerexeta et al.—performed feature selection and reported the findings [7]. They noted that trends in weight and SpO2 were important, blood pressure and HR features, and some obtained with questionnaires, including walking and FOH. Blood presssure, HR features and physical activity (expressed as energy expenditure in our work and as walking in the work by Kerexeta et al.) are common in our and their work, while other differ. The differences are partially due to what data collected (no SpO2 in HeartMan data, and no temperatures, humidity and galvanic skin response in the data of Kerexeta et al.), and partially for other reasons (weight was not particularly important in our experiments).

Variations in patient adherence to telemonitoring protocols and differences in data quality could affect the reproducibility of the results, as models may need to adapt to these factors differently depending on the characteristics of new datasets. In the future, improvements in data collection require attention. Both of the datasets had many missing values and days that were not consecutive. This could help in implementing a new feature such as FOH from yesterday, or any similar feature that gives information about the patient’s previous day. In our experiments, the FOH from yesterday proved to be useful in the Chiron-Transformed dataset. Both Chiron and HeartMan datasets provided satisfactory results, meaning that maybe in the future, with some personalization, an application helping CHF patients to improve their FOH with the help of a predictive model could be brought into the real world. Taking a broader view, we believe that telemonitoring can be very useful if methods that can interpret the large amounts of data collected this way are developed to sufficient maturity for practical use. However, in order to acheive this, the medical community and equipment manufacturers need to settle on a set of variables to be monitored, so that different studies yield comparable data. With decreasing costs and increasing quality of telemonitoring equipment, the set of variables can be quite rich and should certainly include easy-to-measure but often overlooked variables such as ambient temperature and humidity. Then it is critical that researchers combine multiple datasets and that models developed on some datasets are validated on others, as this is the best way achieve confidence in their accuracy. The work presented in our paper is a step in this direction. Furthermore, since both improving FOH and preventing hospitalizations are important objectives of cardiovascular disease management, and—as noted earlier—they can be be accomplished using similar data and methods, they should probably be studied together in the future, and eventual practical applications should tackle both.
